# Development of a conceptual framework for a new patient-reported outcome measure for pain in women following mesh surgery for pelvic floor disorders: a qualitative study

**DOI:** 10.1007/s00192-022-05425-w

**Published:** 2022-12-20

**Authors:** Maisie Ralphsmith, Susannah Ahern, Joanne Dean, Helen E. O’Connell, Rasa Ruseckaite

**Affiliations:** 1 Department of Epidemiology and Preventive Medicine, Monash University, Melbourne, Victoria 3004 Australia; 2 Department of Surgery, University of Melbourne, Melbourne, Victoria 3010 Australia

**Keywords:** Pain, Patient-reported outcome measures, Pelvic floor disorders, Pelvic floor procedures, Pelvic organ prolapse, Stress urinary incontinence

## Abstract

**Introduction and hypothesis:**

The Australasian Pelvic Floor Procedure Registry (APFPR) collects both clinical and health-related quality of life (HRQoL) data on women undergoing surgery using a prosthesis such as mesh for pelvic organ prolapse (POP) and stress urinary incontinence (SUI). The registry lacks a suitable instrument to assess pain in women following mesh surgery for SUI and POP. This qualitative study describes the views on pain following mesh surgery in women and clinicians through the development of a conceptual framework, which may inform the development of a new instrument for the APFPR.

**Methods:**

We conducted semi-structured interviews with women following mesh surgery for POP and SUI (*n*=17) and clinicians (*n*=6) in Victoria, Australia. We sought to reveal aspects of any sort of pain after a pelvic floor procedure. Interviews covered sensation, region, continuity of pain, triggers, and the mode and method of administration for a new pain-specific patient-reported outcome measure. Data were analysed using thematic analysis.

**Results:**

We identified the important components of pain felt by women with POP and SUI after surgery using mesh. From the seven themes outlined, a conceptual framework was developed compiling related components of pain into six specific domains.

**Conclusions:**

This study identifies the important components of pain felt by women following mesh surgery. It is hoped that the development of a pain-specific PROM, as supported by clinicians, will assist in the timely and appropriate diagnosis and management of POP and SUI.

**Supplementary Information:**

The online version contains supplementary material available at 10.1007/s00192-022-05425-w

## Introduction

Pelvic floor disorders (PFDs) involve dysfunction of the urethra and support structures within the pelvic floor [1]. These include stress urinary incontinence (SUI) and pelvic organ (POP) and associated vaginal wall prolapse. The International Urogynecological Association (IUGA) and International Continence Society (ICS) define SUI as the involuntary loss of urine on effort or physical exertion [2], and POP as the descent of one or more of the anterior vaginal wall, posterior vaginal wall, the uterus, or the apex of the vagina [2]. In Australia up to 50% of women are affected by SUI and 9% are symptomatic for POP [3], with a 20% lifetime risk of receiving a pelvic floor reconstructive procedure. Until recently, of the surgical interventions for SUI and POP, it was estimated that approximately 25% involve the use of a mesh product [4]. Following several international health inquiries, thousands of women who have undergone mesh procedures designed to relieve them of the condition have reported adverse events, such as chronic pain due to extrusion of mesh into the vagina [5]. A failure to assess pelvic pain in a systematic fashion post-surgery has contributed significantly. Owing to surgical complications and other presenting symptoms, women with SUI and POP can have significantly impacted health-related quality of life (HRQoL) [5, 6]. As HRQoL is subject to one’s experience and personal beliefs, it is best described by patients themselves [7] through patient-reported outcome measures (PROMs).

The Australasian Pelvic Floor Procedure Registry (APFPR) [8] collects clinical and HRQoL information on patients undergoing surgery for POP and SUI that involves a prosthesis such as mesh. The registry aims to identify and investigate data regarding severe adverse effects experienced by women from procedures involving mesh for pelvic floor surgery [4]. The APFPR is currently implementing PROMs including instruments that are incontinence specific, as well as those that measure sexual function and patient global impression of improvement [9], but does not have an accurate condition-specific instrument that measures pain. This implementation may identify post-operative complications associated with pelvic mesh [10]. A few PROMs that measure pain exist; however, they are not suitable for women with POP and SUI because they lack specificity and fail to recognise the full impact of significant pain arising from these procedures [10]. The addition of a specific instrument to measure pain in the APFPR may add to information regarding the safety of mesh procedures [9] by ensuring that the experiences that are important to women are captured [11]. In order to assess the pain of women after mesh surgery for PFDs, we must endeavour to understand what aspects of pain they experience.

We conducted a qualitative study to understand the views and perceptions of women following mesh surgery for POP and SUI, and their clinicians, on pain. The aim of this project was to develop a conceptual framework defining the important elements of pain as identified by women with PFDs, including preoperative pain where it exists, post-operative pain, or pain due to post-surgical complications. This publication is the first in a series of steps for the addition of a suitable pain condition-specific instrument in the APFPR.

## Materials and methods

### Study design

We used qualitative phenomenological methods to seek to understand personal perspective and meaning, and conducted individual semi-structured telephone interviews to gather data [12]. This approach, while focusing on particular aspects of a topic, does not confine participants to specific response categories defined in advance by researchers, and is particularly appropriate when there is limited evidence for a phenomenon [12].

### Sampling and participant recruitment

Convenience sampling was used through an advertisement that included a brief outline of the study, a contact phone number and a study email. Women participants were recruited through social media via Facebook. Pelvic floor support groups on Facebook were contacted to request they post our advertisement on their page. Women 18 years of age and older who had pain following mesh surgery for POP or SUI were recruited for this study.

Clinicians were health professionals providing services for women with PFDs, and included pelvic floor surgeons, urogynaecologists, women’s health physiotherapists, and research nurse assistants. We attempted to balance the demographic profile of participants by recruiting individuals across different age ranges and specialty types [13].

Potential participants who expressed interest in the research were sent an explanatory statement describing the study. It was not possible to establish how many participants saw the invitation to participate but decided not to volunteer. No participants dropped out of the research. Where possible, written consent was obtained, otherwise recorded verbal consent was attained during telephone interviews.

### Data collection

Researchers developed a semi-structured interview guide based on a previously conducted systematic review [10] and acceptability study [9] to understand views and perceptions on pain following mesh surgery from women with POP and SUI and their clinicians. The interview guide has been included as Electronic Supplementary Material. We explored the sensation of pain, region of pain, continuity of pain, pain triggers, and reflections on the administration of a new PROM. Telephone interviews were conducted between May and July 2021.

All interviewees were fully informed about the study purpose and confidentiality procedures. This was important for the interviewers to establish trust and provide a setting where the participants felt that they could speak freely [14]. All interviews were conducted in a private environment on the phone. Follow-up questions and prompts were used to obtain rich data and all participants were offered the opportunity to review transcripts. Interviews ranged between 24 and 68 min, with an average of 43 min.

### Data analysis

Using NVivo software, we analysed data using thematic analysis [15]. One researcher undertook coding line by line, sorting phrases into categories. These were combined into larger initial themes. Themes were defined and named by merging common elements, largely following the topic guide; however, new categories emerged from the data. To ensure the rigour of coding, a second researcher individually coded 10% of the transcripts. The codes were cross checked, and differences resolved in discussion. These themes were negotiated among all members of the research team, and the initial coding framework was created.

Based on the findings of our systematic review [10] and themes arising from analysis of the interview transcripts, a conceptual framework for an ideal pain instrument for the APFPR was developed. The findings were summarised into a comprehensive diagram and domains from the analysed themes were synthesised, where elements of pain important to women following mesh surgery for POP and SUI and their clinicians were described [16].

## Results

The final number of participants included 17 women and 6 clinicians. The mean (SD) age of women was 57 (8.71) years. Of the women interviewed, 5 (29%) had surgery for POP, 9 (53%) had surgery for SUI, and 3 (18%) had surgery relating to both SUI and POP disorders, all using a prothesis such as mesh. All women suffered pain following surgery using mesh. The mean (SD) age of clinicians was 53 (8.16) years. The specialty of clinicians included 3 urogynaecologists, 2 women’s health physiotherapists, and 1 research nurse assistant. Three of these clinicians were female and three were male. Tables 1 and 2 describe participant characteristics.

**Table 1:** Characteristics of women

Participant ID	Age	Disorder	Complication following surgery	Type of mesh surgery	Time between last surgery and interview (years)
P1	49	SUI	Extrusion and pain	TVT	11
P2	59	SUI	Pain following mesh removal owing to recurrent incontinence	Mini-sling (MiniArc)	10
P3	54	POP	Pain–cause unknown	TFS	8
P4	77	POP	Pain–cause unknown	TFS	6
P5	54	SUI	Pain–cause unknown	TVT-O	7
P6	55	SUI	Pain–cause unknown	TVT-O	7
P7	71	POP	Extrusion and pain	Mesh laparoscopic hysteropexy	8
P8	50	SUI	Extrusion and pain	Mesh for incontinence owing to bladder infections	3
P9	45	SUI	Pain following partial removal of mesh owing to bleeding	TO sling (Monarc)	5
P10	53	SUI	Extrusion and pain	TVT	7
P11	54	POP	Extrusion and pain	TVT	15
P12	54	SUI	Pain–cause unknown	TO sling (Monarc)	12
P13	55	SUI and POP	Pain–cause unknown	TVT and mesh rectopexy for rectocele	8
P14	46	SUI	Pain following mesh removal owing to recurrent incontinence	TVT	6
P15	65	SUI and POP	Pain–cause unknown	TO sling (Monarc)	9
P16	64	POP	Pain–cause unknown	Sacrocolpopexy mesh	14
P17	65	SUI and POP	Pain–cause unknown	Sacrocolpopexy mesh for “global” prolapse and Burch colposuspension for SUI	15

*POP* pelvic organ prolapse, *SUI* stress urinary incontinence, *TFS* tissue fixation system, *TO* trans-obturator, *TVT* tension-free vaginal tape, *TVT-O* tension-free vaginal tape-obturator

**Table 2:** Clinician characteristics

Participant ID	Years in practice	Speciality	Procedure type or role
C1	22	Urogynaecologist	Both mesh and non-mesh for POP and SUI
C2	26	Urogynaecologist	Both mesh and non-mesh for POP and SUI
C3	17	Research nurse assistant	Background in pelvic pain, manages a call-back service to those who had suffered complications post-surgery for POP and SUI
C4	10	Women’s health physiotherapist	Treats women’s health problems, including pelvic floor disorders before and after surgery
C5	20	Women’s health physiotherapist	Assesses and provides conservative management of women post-POP/SUI surgery
C6	27	Urogynaecologist	Both mesh and non-mesh for POP and SUI

### General findings

The themes that were discussed from the topic guide included sensation of pain, region of pain, continuity of pain, pain triggers, pain management and relief, and reflections on the administration of a new PROM. A new theme that arose during interviews involved comorbidities and other complications that may interfere with interpretation or causation of pain experienced post-SUI and/or POP mesh surgery. The women interviewed described components of pain associated with the time course of their PFD procedure.

Each theme is discussed in detail below. Table 3 summarises the major themes along with their subthemes. The seven themes were reduced to six domains, with the “reflections on the administration of a new PROM” being omitted from the final diagram, although still important to consider in any implementation of a new instrument.

**Table 3:** Summary of major themes and their subthemes

Major theme	Subtheme	Women: frequency of mentions		Clinician: frequency of mentions	
Sensation of pain	Sharp, stabbing, shooting	13		6	
	Aching, throbbing	16		2	
	Burning	14		3	
	Numb, tingling, cold	7		2	
	Pulling, dragging, ripping	4		3	
	Cramping, spasm	5		1	
	Electric shock	3		2	
Region of pain	Leg, thigh, toe	18		4	
	Groin	13		4	
	Pelvis or hip	12		2	
	Genitalia	8		4	
	Abdomen	9		2	
	Back and spine	7		4	
	Buttock	10		1	
	Upper body	6		2	
Continuity of pain	Constant	15		4	
	Intermittent	9		1	
Pain triggers	Surgery involving the implant or explant of mesh	Implant 14	Explant 15	Implant 1	Explant 4
	Activities and movements such as walking, sitting, or driving	17		5	
	Intercourse	8		6	
	Sleep	8		2	
Reflections regarding a new instrument and its administration	Suggestions or extra thoughts on a new PROM and its administration	19		6	
	Method of administration	16		6	
	Contact time	10		4	
Comorbidities and other complications that have an impact on patient pain, or cause pain on its own	Pain syndromes such as fibromyalgia and chronic pain	6		6	
	An infection, reaction, or foreign body response to the mesh implant	4		4	
	Other pelvic disorders, such as vulvodynia or bladder irritability	1		3	
	Anxiety and stress disorders had previously, or from previous life events, or because of mesh complications	3		3	
Pain control strategies and pain relief	Medication	10		2	
	Natural “remedies” including a change of diet or use of marijuana and CBD	10		0	
	Mindfulness and meditation	6		1	
	TENS machine, electric current machine	2		1	
	Therapy such as a psychologist	4		3	

### Sensation of pain

Many women described similar sensations of pain. The most frequently reported words to describe the feeling of pain included “sharp, stabbing or shooting”. Fifteen (79%) women mentioned this sensation upon being asked to describe what their pain feels or felt like: *“It was a sharp, stabbing pain, like a serrated knife cutting through you” (P2)*. Many women also talked of an “electricity” feeling: *“I would feel my heartbeat, and then there was this wave of incredible pain in my abdomen and all the way down my leg just like an electric shock” (P16).* One clinician stated that this electric pain was nerve pain, a different sensation from sharper pains: *“That nerve entrapment pain, they’re the ones they describe as burning or shooting or like a knife, like an electric pain. They’re the classic things that go with nerve pain” (C2).*

### Region of pain

There were different locations where pain was experienced, with the most frequently reported regions being in the leg, thigh, or toes; groin; pelvic or hip area; and genitalia: *“Her symptom was burning pain on the left side of her inner labia” (C1).*

Fifteen (88%) women mentioned pain that was manifesting down the leg or thigh owing to adverse events after implant or explant of mesh, 9 (60%) of which had mesh surgery for POP and 6 (40%) for SUI. One woman who had mesh implanted for mild incontinence started to feel pain in her legs a few years post- surgery: *“Down [the] leg, but into the bones in my legs and around the side of my knee, which is strange” (P12).* In contrast, another woman who had mesh for uterine prolapse felt pain in the buttock and groin area straight away upon waking from surgery: “*It felt like somebody had speared me through the buttock and the groin” (P3).* One clinician described a woman’s pain felt in the perineum after surgery as an adverse event: *"She had a reaction to something, to the mesh … And that caused … pain” (C1).*

### Continuity of pain

Women were asked to describe the continuity of their pain. A “constant” pain was mentioned nearly twice as many times compared with an “intermittent” pain: *“I had lower abdominal pain as well… the abdominal pain was all the time” (P2).* An intermittent pain was often attributed to “sharp” or “stabbing”: *“It’s not all day. So it will just come, it just comes and goes…—it’s just a really sharp, sharp pain” (P8).* In contrast, the continuous pain was often attributed to “burning”, “throbbing” or “aching”. One participant stated that following the extrusion of mesh into the vagina, *“This throbbing type sort of feeling is pretty much always there” (P1).*

### Pain triggers

Some women found it difficult to find the words to describe their pain, so often recalled events that triggered it. These events are illustrated in four subthemes: surgery; activities or movement; intercourse; and sleep. Clinicians confirmed that common movements triggered pain: *“Anything, walking, daily living. Some of them can’t get to the letterbox and back” (C5).* Many women attributed pain post-surgery, relating to either the implant or explant of mesh, or to fixing mesh that had eroded into the vagina. After surgery to fix her prolapse with mesh, one woman stated, *“straight away it felt abnormal. It was very, very painful” (P15).* However, 11 women (65%) said that their pain was relieved after having parts or all of their mesh removed: *“The pain actually almost disappeared” (P11)* and *“I was able to get some relief by having the mesh out” (P5).*

### Comorbidities and other complications

There were other reported complications that may obfuscate pain, grouped into subthemes: pain syndromes; other pelvic disorders; infection or reaction; and mental health disorders. One clinician noted: *“Many of these syndromes are included in that cluster of what we could call ‘central sensitisation’ in chronic pain syndromes” (C4).* Moreover, women suffered from a variety of other syndromes: *“I’ve been diagnosed with chronic pain, fibromyalgia, allodynia” (P16).* It seemed to be difficult for clinicians to discern whether the pain is attributed to the mesh itself when conflated with comorbidities. One clinician stated: *“some women have developed pain with an implant in place where it’s really not related to the implant” (C6).* A few women (18%) also mentioned the presence of anxiety, or suffering from depression, and the impact pain has had on their mental health. One woman said, *“I’m trapped in a corner. No-one can help me, and I can’t live like this” (P5).*

### Pain management and relief

A variety of techniques were used by women to relieve pain. These included: medication, meditation, natural remedies, pain relief machines, and psychological therapy. Eight (47%) women relied on (or have had to in the past) strong medication, even after having their mesh divided or partially and fully removed: *“I’m still on gabapentin, which is very strong pain medication” (P5).* The benefits of meditation were highlighted by 7 women (41%), who undertook different approaches such as using phone applications like *“Smiling Mind Meditation” (P4).* Others employed an alternative approach, including medicinal marijuana and cannabidiol (CBD) oil. One woman whose mesh for incontinence had eroded into her vagina says she *“self-medicate[s] with marijuana… Because [she doesn’t] want to get addicted to painkillers” (P1).* Alternatively, another woman who *“live[s] on a TENS machine” (P4)* owing to eroded mesh for prolapse, also mentioned that she is seeing a pain psychologist to help her to manage the pain.

### Mode and method of administration of a new PROM

Upon asking questions about a new instrument, women had many suggestions for elements of pain that they deem important: *“I think what’s important to know is … pain associated with things like going to the toilet, pain associated with sex” (P5).* A clinician envisioned that an addition such as a diagram in the PROM would enable women to easily pinpoint the region of pain: *“You can put a little diagram of the perineum and say, ‘Where is the pain?’” (C1).* Although 17 participants (74%) preferred an electronic mode of administration, many expressed their thoughts on effective ways, with one woman stating, *“I’m not great with computers” (P5).* Clinicians were also divided in their opinion on the administration of other PROMs: *“I’ve got a lot of older patients … They won’t go online to do it” (C2).* Whereas some clinicians preferred the internet: *“You want a digital format, I think” (C1)*.

### Frequency of administration of a new PROM

There were varying attitudes from participants toward the contact time of a new PROM. Not every participant had an answer to this question as they merely did not know. Two clinicians out of 4 (50%) stated that 3-month increments would work best, whereas 1 clinician (25%) preferred 6–8 weeks and 1 (25%) suggested an annual follow-up. One clinician thought: *“six weeks is probably too early, but it’s not a bad idea to get the six-week data, because it tells people how much pain goes away” (C1).* Another clinician expressed: *“Probably not before three months quite honestly” (C2).* Three of 7 women (43%) also thought 3-month increments, 2 (29%) suggested every 6 months, 1 (14%) thought once a month, and 1 (14%) suggested as often as needed.

### Conceptual framework

Based on the findings of these qualitative interviews, we developed a conceptual framework describing what is important to women with PFDs in terms of pain.

**Fig. 1:**
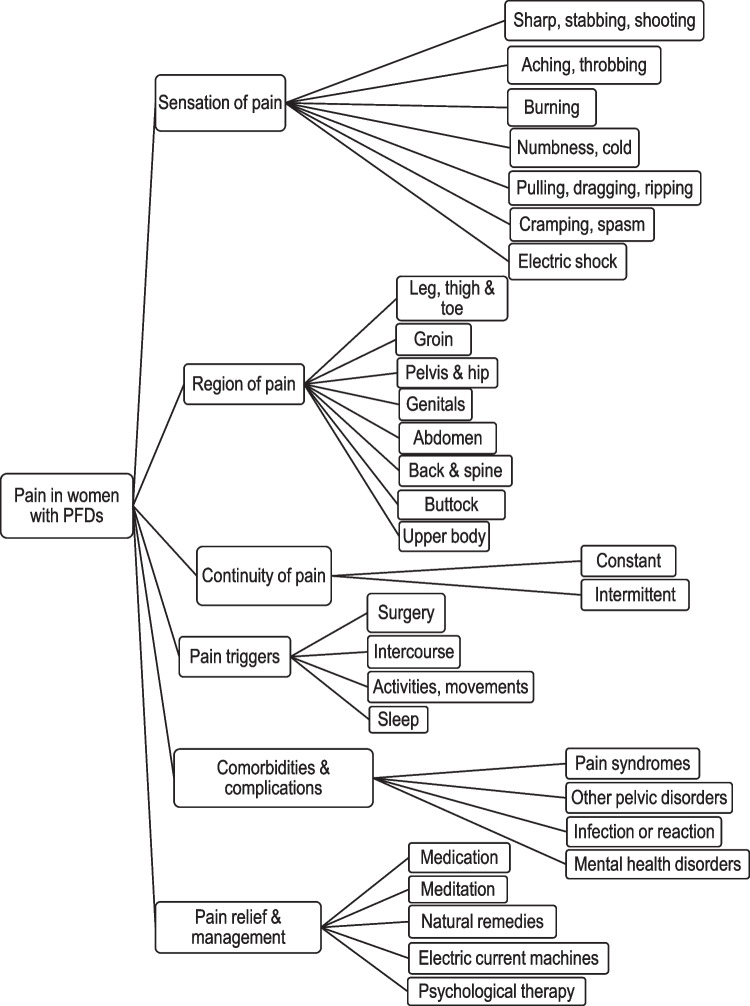
Conceptual framework describing important components of pain for women with PFDs

Figure 1 depicts the conceptual framework that consists of the following domains:

1. Sensation of pain
2. Region of pain
3. Continuity of pain
4. Pain triggers
5. Comorbidities and other complications
6. Pain relief and management

Each domain consists of smaller categories or subdomains. Sensation of pain refers to the feeling of the pain itself and contains subdomains: sharp, stabbing or shooting; aching or throbbing; burning; numbness or cold; pulling, dragging, or ripping; cramping or spasm; and electric shock. Region of pain refers to the location throughout the body and consists of subdomains: leg, thigh, or toe; groin; pelvis and hip; genitals; abdomen; back and spine; buttock; and upper body. Continuity of pain contains two subdomains: intermittent or constant. Pain triggers refer to incidents that coincided with an onset of pain and contains four subdomains: surgery; activities or movements; intercourse; and sleep. Comorbidities and other complications refer to contributing factors experienced by women that may worsen or affect pain owing to their PFD or mesh and involve four subdomains: pain syndromes; infection or reaction; other pelvic disorders; and mental health disorders. Finally, pain relief and management describe how women or clinicians dealt with such pain and have five subdomains: meditation; medication; natural remedies; electric current machines; and psychological therapy.

## Discussion

To our knowledge, this was the first qualitative study to assess and describe what women with POP or SUI deem important when it comes to pelvic floor-related pain following mesh surgery. Outside Australia, existing pelvic floor procedure registries capture important details on the type, number and outcomes of these surgeries [17]. In Australia, there is increasing interest in the collection of PROMs within clinical registries. However, a lack of guidance remains when it comes to which relevant outcome measures to include in national clinical registries for women receiving surgery for POP and SUI [18]. This reinforces the need for the female voice to inform researchers on what is important. Our previous research has identified that the instruments included in pelvic floor registries are not particularly helpful for women following surgery for POP and SUI [9, 10]. It remains a complex situation as pain may exist because of a woman’s original PFD; typical post-operative pain; or pain following mesh surgery due to complications. A pre-existing chronic pain condition prior to PFD surgery is known to be associated with a higher propensity for postoperative pain and pain after mesh removal surgery. Therefore, defining what is important for women with PFDs in terms of pain is imperative as they possess experiential knowledge on their own health in a field where many concerns have felt unheard in the past [19]. Uncovering this perspective is also important following inquiries into pelvic mesh, which led to its subsequent regulation and restriction of use, after thousands of women internationally have suffered from complications including pain [20]. It is hoped that further research will aid in investigating pain to identify pelvic mesh issues more quickly. This qualitative study calls attention to the different attributes of pelvic floor-related pain felt by women. “Sensation of pain” is therefore an important component to consider for an ideal instrument to measure pain, as women feel different sensations. Other QoL instruments have failed to include a domain like this. The five-level EuroQol-5 Dimension (EQ-5D-5L) has just one item to capture pain and other aspects of discomfort [21]. Other studies have found that the use of a single-item question creates more problems than a composite measure [22]. Further, pain PROMs such as the Numeric Pain Rating Scale (NPRS) have been evaluated for use in the registry but women were not clear on the pain they were asked to describe [9]. Other pelvic floor PROMs such as the Pelvic Floor Disability Index 20 (PFDI-20) measure the extent to which symptoms “bother” patients but fail to explore the constancy of pain [23], a potential barrier to understanding the pain itself.

Our study reveals that pain manifests in different locations in women who have undergone mesh surgery for POP or SUI. One reason for this is because mesh can contract, break down, and erode locally, e.g. to the groin or pubic bone, affecting nerve function in the thigh or the organs directly such as the urinary tract or vagina [24]. Thus, there is a need for a pain instrument to encompass relevant extended potential areas of pain in women following PFD surgery.

Determining pain as intermittent or continuous is another valuable factor in information collected around pain in order to understand the underlying pathophysiology (e.g. throbbing pain suggests association with vascular structures). Uncovering pain patterns and progressions may assist in delivering more timely diagnosis and care for these patients.

The “pain triggers” domain in the conceptual framework illustrate that many women with pain following POP or SUI surgery think that it is important to measure the activities that produce pain. This information could provide clinicians with ample detail of when and how pain initiates, informing the best quality of care for women going forward following mesh surgery [25]. By inquiring specifically about certain pain triggers such as those discussed in our findings, a new ideal instrument in the APFPR could inform more targeted care.

Our findings highlight the breadth of pain symptoms experienced by these women, and therefore the importance of a full assessment to properly assess potential pain causes and sequelae. Previous studies demonstrated that poor pain assessment is one of the most problematic barriers to achieving a good-quality diagnosis and pain management [26].

The “complications and other comorbidities” domain sheds light on other predisposing factors or previous medical histories of women with POP or SUI that could impact their pain. Women thought it very important for clinicians to understand such dynamics of the pain experience to get an overall picture of pain. No other pain-related PROM for PFDs collects this type of information for this population.

Previous literature has identified that varying methods of administration of an instrument may be required to maintain a high PROM response rate [27]. This variability was reflected in the semi-structured interviews, with women and clinicians divided in their views on administration. Different methods of administration may be suitable for different demographics.

Many clinicians highlighted the ease of transferring responses to a registry when using electronic PROMs. Recent literature has also outlined how electronic dissemination allows for seamless capturing of PROMs data into electronic health records [28]. Other reported benefits of electronic collection of PROMs includes a faster completion time and higher response rates [29].

The mode of administration of any new PROM should vary between patient populations. Franklin et al. [30] provide a framework for the collection and use of PROMs in learning health care systems that suggests administering PROMs pre- and post-surgery, although the timing and frequency of administration is likely condition specific.

The strength of this study is its use of qualitative analysis, a methodology enabling an in-depth exploration of the experience of pain. The adoption of a qualitative descriptive study design uncovers the perspective of the participant exclusive of interpretation from outsiders such as clinicians. Another strength includes the large sample of women, which ensured that the responses were more likely to be generalisable. All women in this study experienced pain following mesh surgery. We reached data saturation when responses started to repeat themselves, and this was after interviewing 6 clinicians and 17 women. A strength of this study also lies in the diversity of clinician speciality, where data have been gathered from different vocational areas allowing for a more comprehensive clinician voice.

A limitation of this study may include the narrow spectrum of post-surgical complications. Complications included pain due to (partial) removal of a prosthesis such as mesh, pain where the cause was unknown, and pain due to extrusion. Many of these experiences were attributed solely to mesh complications. Women in this study may not have had the “typical” or “usual” post-operative pain experience, and this may be limiting the type of patient experiences we included in this study.

A second limitation of this study may include the participant recruitment method. As participants were selected through social media and advisory consumer groups, and volunteered to participate, it is possible that selection bias could have occurred. Despite aiming for the most representative group of women and clinicians, this is something that may have had an impact on the results.

Another limitation of the study may be recall bias. Because women were asked to describe the time course of their pain from the time of surgery, retrospectively, the completeness and accuracy of the information may have impacted our results.

This study provides insight into the specifics of pain that impact women following mesh surgery for POP and SUI, which is currently not being accurately measured by any existing instrument. We now understand elements such as the sensation, region, continuity, triggers, and other complications of pain, as well as how women manage their pain. The women in this study describe components of pain that are the types of complications important for a new PROM to measure. The domains outlined in the conceptual framework may serve as a reminder of the importance of pain in assessing safety issues for women after mesh-related procedures.

## Conclusion

This study provides qualitative evidence of the components of pain that are important from the perspective of women following PFD surgery. We developed a conceptual framework of pain in women following PFD surgery using mesh, including domains of sensation of pain, region of pain, continuity of pain, pain relief and management, pain triggers, and complications and other comorbidities. These domains may provide a basis for the development of a new pain- specific instrument for the APFPR.

## Supplementary Information

ESM 1 (PDF 78 kb)

## Data Availability

Transcripts for the data are available from the corresponding author on reasonable request.
