# Supplementary material for: Development of a conceptual framework for a new patient-reported outcome measure for pain in women following mesh surgery for pelvic floor disorders: a qualitative study
Source: Int Urogynecol J. 2022 Dec 20;34(7):1541–50. doi: 10.1007/s00192-022-05425-w (PMC10287803; doi:10.1007/s00192-022-05425-w)
Supplement: Supplementary file 1 — (PDF 78 kb) [file 192_2022_5425_MOESM1_ESM.pdf]

Online Resource: Interview Guide

Patients

GENERAL

Have you undergone surgery for a pelvic floor disorder?
When was your surgery?
What kind of disorder do you have? Prolapse or urinary incontinence?
What was the procedure that you had done?
Did you experience mesh complications such as erosion of the mesh or infection?
Do you currently have pain or did you have pain? If not now, when did you have pain last?
After surgery, do you believe your pain was typical post- operation pain or was it pain due to a complication with the surgery?

SENSATION AND REGION

What does the pain feel like if you could describe it for me?
Is it only in your pelvic region or is it anywhere else in your body?
Is the pain you feel now the same pain felt before you had surgery?
Do you have fear of pain, fear that you will randomly get pain?
Do you have fear of your pain worsening?
Does it hurt when passing urine?
Do you have to stop movement (walking/ exercising/ jobs around the house) when this pain occurs?
How does it impact your social life?

TIME POINTS

When did the pain start? Did it first start after surgery?
How long after surgery did the pain start?
Does it hurt more now after surgery or did it hurt more before surgery?
In a normal day, at what time points do you feel pain? After or during sex, only when urinating, at the end of the day?
Do you try to relieve this pain? How do you manage- through medication? Anything else? When do you take medication?

DURATION

Is it long lasting or quick, intermittent pains?
How long does it last?
Does it start and stop?
Do you feel pain all the time?

PROM

As you know, I'm a part of the APFPR team at Monash. The registry was set up to collect data on women with pelvic floor disorders. We are looking at developing a new questionnaire or survey that could be delivered to women like yourself to accurately measure pain, because everyone feels pain differently, at different times, and things like that. The aim of this study is to try and understand what women deem important when it comes to their pain.
I'm now going to be asking a few questions about the new survey or questionnaire that we intend on developing to try and accurately capture this pain.

Do you have any suggestions on some questions about pain that should be included?
When or how often do you think one should be contacted to measure their pain?
What do you think the best mode of administration is? Via email? Phone? Internet? On paper?

Clinicians

GENERAL
Do you perform surgery for pelvic floor disorders?
How long have you been a surgeon for?
Is this surgery for POP or SUI or both? / What do you see more of?
How many surgeries do you perform a year?
Do you treat women with typical post operation pain or atypical pain associated with complications from surgery?
What are women's perception pre-surgery? Are they excited in hope of pain being eradicated/ nervous/ worried about pain?

SENSATION AND REGION
What are some of the type of pain that women report before surgery?
What are some of the type of pain that women report after surgery?
How do they describe this pain?
Is it only in their pelvic or vaginal region or is it anywhere else in the body?
Is the pain they feel now the same pain felt before surgery?
Do patients have fear of pain?
Do they liken the pain to menstrual cramps?
Do women say it hurts when passing urine?
Do they have to stop movement (walking/ exercising/ jobs around the house) when this pain occurs?
What are the pain comorbidities that you experience in women reporting their pain?

TIME POINTS
What has been your experience comparing patients' pain before and after surgery?
Are women normally in less or more pain after receiving the mesh implant?
At what time points do they feel pain? After or during sex, only when urinating, at the end of the day?
Do they try to relieve this pain? How do they manage it- through medication? Anything else? At what time points?

DURATION
Is it long lasting or quick, intermittent pains?
In your experience, do patients say that the pain is constant?

PROM
Do you think it would be beneficial to develop a new pain specifically for women with pelvic mesh pain?
Do you expect any negative consequences from this?
What questions should be included?

When or how often do you think one should be contacted to measure their pain? What do you think the best mode of administration is? Via email? Phone? Internet? On paper?
